# Differential diagnoses of right lower quadrant pain in late pregnancy

**DOI:** 10.1093/jscr/rjac200

**Published:** 2022-07-25

**Authors:** Diana L Daume, Pauline M Becker, Katja Linke, Jean-Jacques Ries, Lana Fourie, Jennifer M Klasen

**Affiliations:** Clarunis, Department of Visceral Surgery, University Center for Gastrointestinal and Liver Diseases, St. Claraspital and University Hospital Basel, Basel, Switzerland; Clarunis, Department of Visceral Surgery, University Center for Gastrointestinal and Liver Diseases, St. Claraspital and University Hospital Basel, Basel, Switzerland; Clarunis, Department of Visceral Surgery, University Center for Gastrointestinal and Liver Diseases, St. Claraspital and University Hospital Basel, Basel, Switzerland; Department of Obstetrics and Gynecology, University Hospital of Basel, Basel, Switzerland; Clarunis, Department of Visceral Surgery, University Center for Gastrointestinal and Liver Diseases, St. Claraspital and University Hospital Basel, Basel, Switzerland; Clarunis, Department of Visceral Surgery, University Center for Gastrointestinal and Liver Diseases, St. Claraspital and University Hospital Basel, Basel, Switzerland

## Abstract

We present two patients with right lower quadrant pain during the 36th week of pregnancy. In both cases, the challenges in diagnosing acute appendicitis in late pregnancy is underlined by misleading imaging results, revealing fluid in the lower abdomen, suggesting an appendicitis. Surgery was performed. Pre- and intraoperative gynecological examinations showed no signs of fetal distress. In patient 1, surgery revealed a torsion and necrosis of the right ovary and a 7-cm cyst of the fallopian tube. Open ovariectomy and appendectomy were performed. In patient 2, we saw a perforated appendicitis and cloudy ascites. Histology after appendectomy showed spots of endometriosis and serositis infiltrating into the appendix with signs of perforation at the tip. Patient 1 recovered after a short period of bowel paralysis. Patient 2 needed Caesarean section due to severe deceleration in the cardiotocograph and irregular uterine contractions. The newborn was kept in the neonatal ICU for 10 days.

## INTRODUCTION

Pain in the right lower abdomen occurs as an inconsequential pregnancy-related symptom, but it can also be the first clinical presentation of a diagnosis requiring surgical intervention. During pregnancy, acute appendicitis is the most common complication leading to surgery [1, 2]. Symptoms include nausea, vomiting or fever. Laboratory investigation may reveal a leukocytosis with left shift [3], increased serum bilirubin [4] and CRP. In pregnancy, the clinical presentation is often atypical [5]. As the uterus enlarges, the appendix may migrate upward and with it the localization of pain. Leukocytosis can be pregnancy related [1, 5]. Nevertheless, right lower quadrant pain remains the most reliable clinical symptom of acute appendicitis regardless of the gestational trimester [1]. In doubt, diagnostic imaging (ultrasonography or MRI) should be performed [6, 7]. Because delayed surgical intervention increases the risk of perforation [8, 9], patients with suspected acute appendicitis should undergo prompt appendectomy. We present two cases of right lower quadrant pain during the 36th gestational week and MR-tomographically suspected acute appendicitis. Open surgery was performed in both cases, but the diagnoses deviated from the presumptive diagnoses. Both cases confirm how challenging a surgical diagnosis of abdominal pain in pregnancy can be.

## CASE REPORT

**Figure 1:**
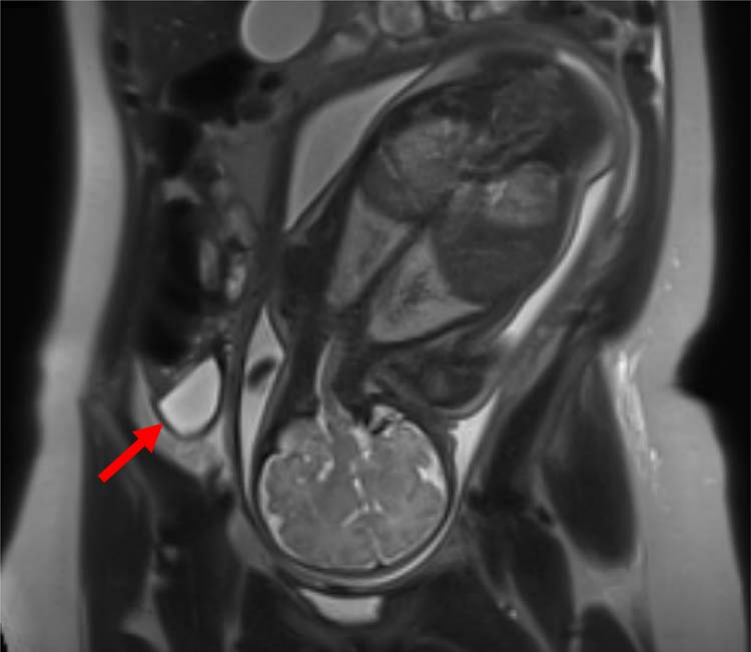
MRI of patient 1 revealing a fluid collection in the right lower abdomen.

Patient 1 (31 years, 35 + 4 weeks of pregnancy) was suffering colicky pain in the right lower quadrant accompanied by symptoms of nausea, emesis, and an episode of diarrhea since the night before. Clinical examination revealed strong pain with guarding upon palpation of the right lower abdomen. Blood tests showed no abnormalities. Pregnancy-related causes were excluded by gynecological examination; cardiotocography and fetal ultrasonography were both physiological. An urinary infection was suspected, so the patient was hospitalized and intravenous antibiotic treatment (co-amoxicillin) initiated. Two days after admission, an MRI revealed a fluid collection in the right lower abdomen. The appendix could not be delineated clearly (Fig. 1).

On the fourth day after admission, serum CRP level rose to 66.8 mg/l. Follow-up ultrasonography detected a mass in the right lower abdomen, suggestive of an abscess. An interdisciplinary decision was made in favor of surgical exploration under prophylactic perioperative tocolysis with Atosiban. Midabdominal horizontal right-sided laparotomy was performed, showing torsion and necrosis of the right ovary and hemorrhagic ascites. The appendix appeared reddened and thickened. The ovary was removed, and incidental appendectomy performed. Histology showed hemorrhagic infarction of the ovary and fallopian tube and a normal appendix. The patient showed a small bowel paralysis, but recovered quickly and was discharged 1 week after surgery without complications for the infant.

Patient 2 (35 years, 35 + 3 weeks of pregnancy) reported pain in the lower abdomen for 4 days. Palpation provoked pronounced pain in the right lower quadrant, rebound tenderness on both sides of the lower abdomen. Blood tests revealed leukocytosis (18.9 × 109/l) and an elevated CRP (42 mg/l). Cardiotocography and fetal ultrasonography were normal; the appendix could not be detected on ultrasonography. MRI showed a distended appendix with an adjacent fluid collection and surrounding imbibition of fat tissue. An open appendectomy was performed and a perioperative antibiotic treatment with co-amoxicillin was started. Perioperative tocolysis with Hexoprenaline was administered. The retrocecal appendix appeared inflamed, and perforated, surrounded by cloudy ascites. However, the histopathological examination showed no signs of appendiceal inflammation, but several spots of endometriosis, triggering serositis.

Showing a rising leukocytosis (20.75 × 10^9^/l) and CRP (291.5 mg/l) on the first postoperative day, this patient presented irregular uterine contractions. Because of the cardiotocography revealing episodes of severe deceleration, an emergency C-section was performed. Cloudy ascites was detected in the pelvis; the amniotic fluid remained clear and a healthy boy (2220 g) was delivered. He recovered quickly after initial respiratory insufficiency (Apgar scores 3, 6 and 9, umbilical artery pH 7,21). Due to detection of *Bacteroides fragilis* in the abdominal fluid, oral antibiotic treatment was continued until 9 days postoperatively.

## DISCUSSION

Acute appendicitis is the most relevant differential diagnosis of right lower quadrant pain in pregnancy, while the likelihood of an acute appendicitis during pregnancy is about 0.1% [2, 6].

However, it remains the most frequent complication, requiring immediate surgery [1]. Pain, leukocytosis, nausea and vomiting, fever, and bowel irregularities can occur during pregnancy, which is why diagnosing appendicitis is challenging and necessitates focused clinical and radiological investigations. Ultrasonography and MRI may help to detect differential diagnoses such as ovarian torsion, cyst rupture, gallstone diseases, nephrolithiasis and pyelonephritis [6]. Once acute appendicitis is suspected clinically and radiologically, any delay of surgery increases the risk of maternal and fetal complications [7, 8]. Owing to the sparsity of research data, antibiotic treatment alone is not a reasonable therapeutic option [10, 11]. Both laparoscopic and open appendectomy can be considered. The advantages of laparoscopic appendectomy seem obvious: fewer overall complications and a shorter hospital stay [12, 13]. On contrary, the open procedure, is associated with lower risk of fetal loss [13, 14] and may be easier to perform at an advanced gestational age. The choice of the procedure should be made carefully in each individual case, taking into account the experience of the surgeon. Overall, the significant increase in risk of fetal and maternal mortality following delayed therapy with appendiceal perforation, outweighs the rates of negative laparotomies performed in pregnant patients [14, 15]. This strong argument in favor of surgery is also reflected in the recommendation to perform incidental appendectomy in case of macroscopic normal appearing appendices, since almost one-third of normal appearing appendices show histological signs of inflammation [16]. Endometriosis, as in patient 2, is a differential diagnosis rarely described in the literature and even more rare being the cause of inflammation [17].

An emergency C-section was performed in patient 2 within 24 h after surgery. Although the data show that the risk of preterm delivery is low [2], it has to be taken into careful consideration.

The most appropriate treatment should ideally be decided in a center with easy access to neonatologists, and close collaboration between abdominal surgeons, obstetricians and anesthesiologists, with the option of C-section at any time.
